# The Role of Cognitive and Perceptual Loads in Inattentional Deafness

**DOI:** 10.3389/fnhum.2016.00344

**Published:** 2016-07-06

**Authors:** Mickaël Causse, Jean-Paul Imbert, Louise Giraudet, Christophe Jouffrais, Sébastien Tremblay

**Affiliations:** ^1^Département Conception et Conduite des Véhicules Aéronautiques et Spatiaux, Institut Supérieur de l’Aéronautique et de l’Espace (ISAE)Toulouse, France; ^2^School of Psychology, Co-Dot Laboratory, Université LavalQuébec, QC, Canada; ^3^Laboratoire d’Informatique Interactive (LII), École Nationale de l’Aviation Civile (ENAC)Toulouse, France; ^4^Centre National de la Recherche Scientifique (CRNS) and Université de Toulouse, IRIT, ToulouseFrance

**Keywords:** inattentional deafness, cognitive load, perceptual load, pupil diameter, neuroergonomics

## Abstract

The current study examines the role of cognitive and perceptual loads in inattentional deafness (the failure to perceive an auditory stimulus) and the possibility to predict this phenomenon with ocular measurements. Twenty participants performed Air Traffic Control (ATC) scenarios—in the Laby ATC-like microworld—guiding one (low cognitive load) or two (high cognitive load) aircraft while responding to visual notifications related to 7 (low perceptual load) or 21 (high perceptual load) peripheral aircraft. At the same time, participants were played standard tones which they had to ignore (probability = 0.80), or deviant tones (probability = 0.20) which they had to report. Behavioral results showed that 28.76% of alarms were not reported in the low cognitive load condition and up to 46.21% in the high cognitive load condition. On the contrary, perceptual load had no impact on the inattentional deafness rate. Finally, the mean pupil diameter of the fixations that preceded the target tones was significantly lower in the trials in which the participants did not report the tones, likely showing a momentary lapse of sustained attention, which in turn was associated to the occurrence of inattentional deafness.

## Introduction

The Air Traffic Control (ATC) environment involves supervisory control of emergency response, and security surveillance. Air traffic controllers must deal with dynamic and cognitively demanding tasks: guiding aircraft through a controlled airspace and optimizing trajectories whilst adhering to minimum distance and altitude separation minima requirement. This task must be completed in the face of temporal pressure, stress, and high-risk decision-making situations. Several research tried to identify the characteristics of the ATC environment that create cognitive demand (e.g., Manning et al., 2002; Loft et al., 2007). Manning et al. (2001) showed that these characteristics include, among others, the total number of aircraft controlled, the number of aircraft changing altitude, and the total conflict alert displayed. Other studies revealed that the dynamic density of the airspace at a given moment accounts for approximately half the variance in workload (Laudeman et al., 1998; Kopardekar and Magyarits, 2003). Although task demand has a strong relationship with workload, this relationship depends on the ATC operator capacity to select priorities and manage its cognitive resources (Loft et al., 2007).

The auditory channel is an essential means for air traffic controllers to exchange information with pilots and other controllers through radio and phone communications. They must also be vigilant and responsive to the occurrence of auditory alarms such as ground collision avoidance alerts or area infringement warnings that have been increasingly integrated into ATC workstations (Cabrera et al., 2005). Given that the auditory modality provides information without requiring head/gaze movements (Edworthy et al., 1991), it is particularly suitable for the transmission of alerts and warnings in emergency situations because perception is not dependent on the direction of gaze at a particular moment (Harris, 2011). However, research in the field of aviation has provided ample evidence that individuals can still remain unaware of unexpected task-relevant and often safety-critical auditory stimuli if deeply involved in demanding tasks (Dehais et al., 2012, 2014; Giraudet et al., 2015b).

Several studies support the notion of a central bottleneck of attention processing (Jolicoeur, 1999; Arnell and Larson, 2002; Lavie, 2005; Dux et al., 2006; Raveh and Lavie, 2015; Wahn and König, 2015) but other works propose modality-specific restrictions of attention (Duncan et al., 1997; Talsma et al., 2006; Martens et al., 2010; Keitel et al., 2013). In accordance with the first view, Tombu et al. (2011) proposed a central attentional bottleneck that includes the inferior frontal junction, superior medial frontal cortex, and bilateral insula that temporally limits cognitive processes such as perceptual encoding or decision-making. In contrast, other studies show support for modality specific limitations by demonstrating that attentional capacity between modalities is greater than attentional capacity within the same modality (Talsma et al., 2006). Furthermore, Martens et al. (2010) showed that an attentional blink is produced only when targets are both presented within the same modality (auditory or visual) but not cross-modally, thus favoring the idea of a modality-specific sensory system rather than a central amodal system. From a theoretical viewpoint, multiple resource theory (Wickens, 1980) posits that there are multiple, independent pools of resources and that tasks that share the same limited resource would interfere with each other but would not affect other tasks that require a different type of resource. For example, Kim et al. (2005), showed that Stroop interference increased when the type of working memory (WM) load overlapped with the type of information required for the target task. At the same time, Stroop interference decreased when the type of WM load overlapped with distractor processing. Beyond this debate between central vs. modality-specific attentional limitations, many studies show that WM load also affects the ability to process visual or auditory environmental stimuli. For example, Sörqvist et al. (2012) demonstrated that brain response to an irrelevant sound decreased as a function of central WM load, induced by a visual-verbal version of the n-back task. In the same way, it has been shown that manipulating the task load of the primary task reduced markedly the sensitivity to auditory distractors during a duration-discrimination task (Berti and Schröger, 2003).

Given the evidence for both sides of the amodal vs. modality-specific debate on attentional capacity, we might postulate the existence of both central limitations in the control of attention and executive control (Rossi et al., 2009), with a key role of the prefrontal cortex (Asplund et al., 2010) and higher-order multisensory cortices (Calvert and Thesen, 2004), and additional capacity limits in modality-specific sensory brain areas (Talsma and Kok, 2001). Such a hypothesis is supported by Vachon and Tremblay (2008) using an attentional blink paradigm. Their results tend to support the idea that attentional limitations are due to a mixture of both modality-specific and amodal resource constraints. Based on the results of Berti and Schröger (2003) and Sörqvist et al. (2012) showing the adverse effect of WM load, as well as similar fundamental works (Wood and Cowan, 1995; Spence and Read, 2003; Lavie et al., 2004; Hughes et al., 2013) and observations in flight and ATC simulators (Dehais et al., 2012, 2014; Giraudet et al., 2015a,b) indicating that a high cognitive load context can lead to the neglect of auditory alerts, we may reasonably postulate that the risk of missed alarms is quite important in complex activities such as ATC.

The high cognitive and perceptual loads typical of ATC operations may consume most of attentional resources, thus reducing the remaining attentional capacity for processing unexpected stimuli such as auditory alarms. This failure to perceive auditory stimuli has been called inattentional deafness (Macdonald and Lavie, 2011; Koreimann et al., 2014). Given the potential impact of inattentional deafness in safety-critical occupations, it is important to understand the factors that promote this phenomenon and to be able to detect its occurrence. When no visual feedback from the operator is available, or when the alarm is triggered by a system, it is almost impossible to interpret human reactions. However, recent studies have found electro-encephalographic indicators of the occurrence of inattentional deafness with diminution of the amplitude of the P300 evoked potential (Giraudet et al., 2015a,b). These results are promising since they allow an offline analysis to test alarm designs and to evaluate the conditions favoring inattentional deafness. However, the online detection of inattentional deafness with ERP is complex under ecological conditions given the low signal-to-noise ratio of the event-related EEG activity. A more robust way for detecting inattentional deafness online is still to be developed, but the ability to predict its occurrence using a physiological measure has excellent potential. With the visual modality monopolizing most of attentional resources, we suggest that recording eye movements while operators are exposed to alarms can inform about their auditory capacity in real time, particularly if they are displaying inattentional deafness. Eye-tracking has already proven very useful for interface design and for usability tests (Goldberg and Kotval, 1999). Several behavioral ocular metrics such as the number of fixations and their duration, the scanpath direction and length, or the switching rate between areas of interest can provide a non-invasive measure of cognitive activity (for a review see, Jacob and Karn, 2003). Evidence suggests that when the eye is free to move, fixation location is strongly correlated with where attention is focused (Findlay and Gilchrist, 2003). But while eye tracking is known to reflect visual cognition, it is uncertain whether ocular behavior could reflect further mental processes beyond basic visual encoding of task-relevant information. Also, the pupil diameter is a classic measure to index cognitive activity and is generally higher in context of high mental workload (Kahneman and Beatty, 1966; Palinko et al., 2010; Peysakhovich et al., 2015) or when the level of vigilance is high (Beatty, 1982). For example, Beatty (1982) showed that vigilance decrement was associated to decreased amplitude of the phasic task-evoked pupillary response during an auditory vigilance task, while tonic or baseline pupillary diameter exhibited no such relationship.

Inattentional deafness is generally studied by varying perceptual load (Koreimann et al., 2009, 2014; Macdonald and Lavie, 2011; Molloy et al., 2015; Raveh and Lavie, 2015), while the effects of variations in mental workload (central demand) are less well investigated (Giraudet et al., 2015b). Importantly, no studies have examined and compared the impact of these two loads on the ability to perceive auditory stimuli. The present study had two main objectives: to further understand how cognitive and perceptual loads impact auditory detection sensitivity, and to assess the possibility of eye-movements and pupil diameter to predict the occurrence of inattentional deafness. Twenty participants performed a realistic ATC simulation task called Laby (Imbert et al., 2014a) while an auditory oddball task was presented. Participants had to react to deviant tones (simulating auditory alarms) by button pressing, as an indicator of their detection of the sound. We separately examined the impact of cognitive and perceptual loads on auditory detection sensitivity with a 2 × 2 factorial design. The cognitive load varied with the number of central aircraft to control, and the perceptual load with the number of peripheral aircraft to monitor. In a previous study also using Laby, we demonstrated that the cerebral response (P300) to deviant auditory tones was diminished when the visual design of Laby was poor (Giraudet et al., 2015a). Also, this study showed that approximatively 6% of the deviant tones were missed in the high cognitive load condition with the poor visual design. To further understand the factors that promote the occurrence of inattentional deafness, in the present study we intended to increase the inattentional deafness rate by using a more engaging and complex version of the Laby. Inducing a high level of missed alarms would enable comparison between the ocular behavior of missed and reported alarms. We hypothesized that: (1) the high cognitive and perceptual load conditions should generate more missed alerts than the low cognitive and perceptual load conditions; (2) increased cognitive and perceptual load should impact ocular measurements; and (3) ocular measurements may predict the occurrence of inattentional deafness.

## Materials and Methods

### Participants

Twenty participants, all students of Université Laval were recruited for this study (Mean age = 23.5 years, Standard Deviation (SD) = 4.2). None had a history of neurological disease, psychiatric disturbance, substance abuse, or took psychoactive medications. They all received full information on the experimental protocol, signed an informed consent and received compensation for their participation in the study.

### Experimental Design

We used a 2 × 2 factorial design crossing two independent variables, cognitive and perceptual loads. The cognitive load was manipulated by the number of central aircraft in the corridor. The low cognitive load condition was the first half of the scenarios, with one aircraft to guide. The high cognitive load condition was the second half of the scenarios, with two aircraft to guide. The perceptual load was manipulated by the number of peripheral aircraft around the corridor (between 5 and 21). The perceptual load was unique for each scenario and the order in which low and high perceptual load scenarios was performed was counterbalanced across participants.

### The Laby Microworld and The Auditory Oddball Task

#### The ATC Task

**Figure 1:**
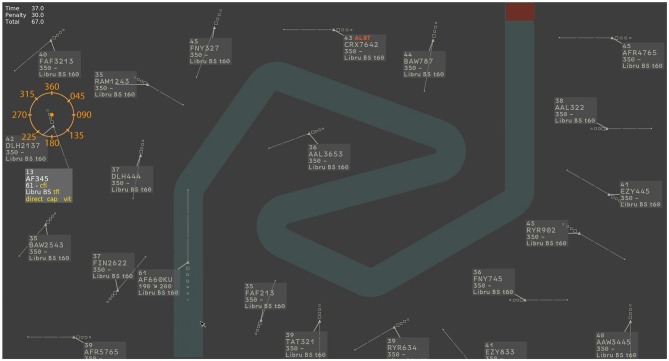
**Screenshot of the Laby microworld simulation.** An example with 21 static peripheral aircraft positioned around the corridor. The central aircraft navigates through the corridor.

The Laby microworld is a functional simulation of ATC, developed to create and evaluate new designs for controller’s visualization. It is built on the main task of guiding aircraft around a route shown on the center part of the screen (light green path). For the first half of the Laby scenario, there was only one aircraft to monitor. In order to increase the main task demand, at the beginning of the second half of the scenario, a second aircraft entered the corridor and participants had to guide both aircraft along the route (Figure 1).

**Figure 2:**
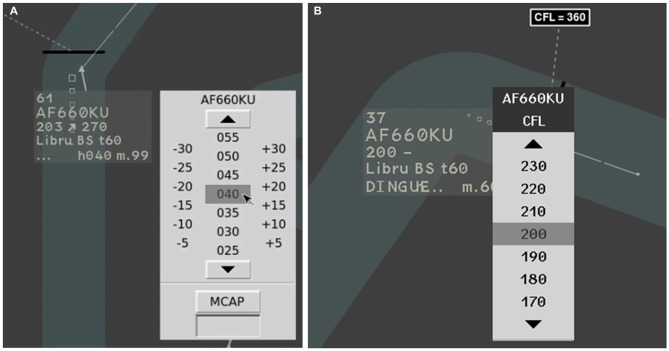
**Zoom on the Laby interface. (A)** The menu used to select the heading of the central aircraft. **(B)** The menu used to select the altitude of the central aircraft. The menus appeared when clicking on the radar label.

In order to maintain the central aircraft within the corridor or to follow altitude instructions, participants had to regularly modify their heading and altitude, using drop-down menus (Figures 2A,B).

**Figure 3:**
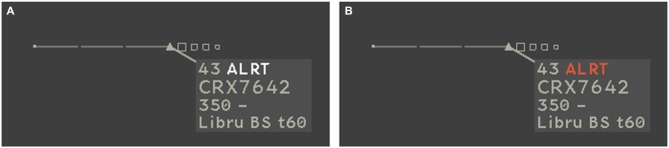
**The visual notification Color-Blink is inspired by operational warnings triggered in Air Traffic Control (ATC) radar screens when minimum separation between aircraft is lost.** The text ALRT switches from white **(A)** to red **(B)** at a rate of 200 ms white/800 ms red.

In addition to the central aircraft, participants had to monitor a set of static aircraft (5 in the low perceptual load condition, 21 in the high perceptual load condition) located around the main aircraft corridor (Figure 1). “Color-Blink” visual notifications were displayed in or around the radar label located in the vicinity of these peripheral aircraft (Figure 3). Color-Blink uses colored text with the word “ALRT” which switches from white to red (see Figures 3A,B). It is used in ATC operational radar visualization for high-priority short-term conflict alerts. The Laby interface design is similar to operational radar visualization, and has been used in a previous study comparing the performance of several visual notifications in peripheral vision (Imbert et al., 2014b). Compared to other enhanced designs, the Color-Blink notification was found to be less salient and had a lower detection rate among controllers (see, Imbert et al., 2014a). We thus selected the Color-Blink notification to increase the overall monitoring effort in the present study. In another study also with Laby (Giraudet et al., 2015a), we showed that a high cognitive load condition of the Laby was associated with 6% unreported ton. Also, as we intended to increase the inattentional deafness rate with a more engaging and complex versions of the Laby in the present study, two modifications were performed. In the present study, there was two aircraft to guide in the high cognitive load condition (one in the previous study). In addition, contrary to the previous studies in which the heading indications to give to the aircraft was already computed by the system and just had to be selected by the participants in a drop-down menus, in the present study, the participants had to mentally calculate the various heading that the aircraft should follow to turn and stay in the corridor. An orange heading indicator was displayed on the top left corner in order to help participants to transform direction into heading values in degree.

Visual notifications were randomly displayed in the radar label located in the vicinity of these peripheral aircraft. Only one notification was issued at a time. The notification disappeared as soon as the participant clicked on the aircraft. If the participant did not react within a given time (5 s), the notification disappeared. Thirty-four visual notifications were displayed in each scenario.

A score was displayed on the top left of the screen. The score decreased for the following three reasons: first, when a participant led an aircraft outside the corridor; second, when he/she gave an incorrect altitude instruction; third, when he/she failed to click on a peripheral notification in the time limit. A deviation in the assigned route resulted in the aircraft crossing the border and initiating a visual alert in the center of the screen. An error in the altitude instructions resulted in the aircraft maintaining its trajectory, with no alert, and continued control. The score aimed to engage the participant in the ATC-like simulation in order to avoid them paying attention to the auditory alarm detection task only. The score was not considered in the analysis. The simulation ended as soon as the first aircraft reached the arrival area (colored red), at the end of the corridor.

#### Auditory Oddball to Simulate an Alarm Detection Task

In parallel to the ATC task, participants had to perform an auditory alarm detection task. Standard pure tones (1000 Hz, 52.5 dB SPL, 500 ms long, probability = 0.8) and deviant pure tones (2000 Hz, 52.5 dB SPL, 500 ms long, probability = 0.2) were randomly played. The tones were not representative of the auditory alerts recently integrated in ATC operations, their frequencies were chosen from previous works (Giraudet et al., 2014, 2015a,b). The mean time window between successive tones was 4.2 s. Participants were told to consider the deviant tones as auditory warnings and to report them as fast as possible by pressing a specific button. The auditory oddball detection task had no impact on the score. The number of auditory alarms (10) was the same in the four experimental conditions. There were ten tones in the first half (with one main aircraft) and ten tones in the second half (two aircraft). In order to increase the sound detection task difficulty, A 42 dB white noise was played continuously during each ATC scenario and the oddball control task. A control condition was also performed by the participant. They only had to react to the deviant auditory tones of the oddball while fixating a cross at the screen.

### Procedure

The whole procedure lasted about 1 h. First, participants were seated comfortably at 60 cm from the 30″ screen in a sound-attenuated room with their right hand on the computer mouse and their left hand on the auditory alarm button. Second, they completed a training phase of 5 min to familiarize with the Laby microworld software, i.e., enter correctly path and altitude instructions by the drop-down menus, acknowledge visual notifications, and report deviant tones. After the training, the eye tracker was calibrated and participants completed the four ATC scenarios. Between scenarios, the eye tracker was recalibrated. Finally, participants performed the auditory oddball control task.

#### Eye Tracking Measurements and Data Processing

Continuous eye tracking was performed with a Tobii T1750 during the four ATC scenarios. The signal was recorded at a sampling rate of 300 Hz. For all eye movement analyses, the threshold to detect a fixation was set at 100 ms and the fixation field corresponded to a circle with a 30-pixel radius (equivalent to 1.15° of visual angle when seated at a distance of 50 cm). The position of both eyes on the screen was recorded. Data analysis was performed using MATLAB 7.1 (The Mathworks). Heatmaps visualizations of the distribution of fixations were generated using the open source software Open Gaze and Mouse Analyzer (OGAMA; Vosskühler et al., 2008).

#### Statistical Analysis

The impact of cognitive and perceptual loads on the accuracy to the central aircraft guiding task, peripheral notifications detection rate and missed auditory stimuli (rare tones) were analyzed. We also calculated the mean fixation duration on each of the four whole scenario and the mean duration of the fixation time that preceded the onset of a deviant tone (time-locked analysis) as well as the mean pupil diameter of this fixation (averaged on both eyes). Statistical analyses were performed using Statistica 10 (StatSoft^©^). Differences between the experimental conditions were investigated with the use of within-subjects analysis of variance (ANOVA) followed by *post hoc* testing (Tukey’s honestly significant difference, Tukey HSD). We finally computed a multivariate logistic regression in order to further determine the variables that predicted inattentional deafness.

## Results

### Effects of Cognitive and Perceptual Loads on Performance to The ATC Task

**Figure 4:**
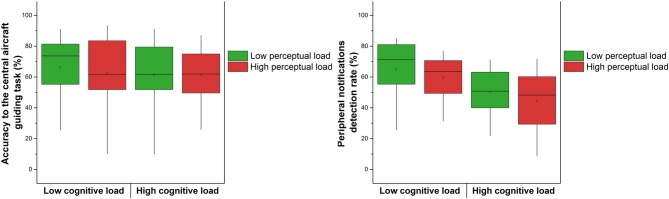
**Correct responses to the central aircraft guiding sub-task according to the levels of cognitive and perceptual loads.** Validation of the peripheral aircraft sub-task according to the levels of cognitive and perceptual load. The square in the center of the boxes represent the mean, the horizontal line in the center of the boxes represent the 50th percentile (median), the end of the boxes represent the 25th and 75th percentiles, and the whiskers represent the 5th and 95th percentiles.

We examined if the performance in the ACT task depended on cognitive and perceptual loads, see Figure 4. The 2 × 2 (cognitive load × perceptual load) repeated measures ANOVA showed no significant effect of the cognitive and perceptual loads on the accuracy to the central aircraft guiding task (respectively, *F*_(1,19)_ = 0.86, *p* > 0.05, $\eta_{p}^{2}$ = 0.04; *F*_(1,19)_ = 0.64, *p* > 0.05, $\eta_{p}^{2}$ = 0.03). The interaction term was not significant (*F*_(1,19)_ = 0.37, *p* > 0.05, $\eta_{p}^{2}$ = 0.01). Regarding the peripheral notifications detection rate, the 2 × 2 (cognitive load × perceptual load) repeated measures ANOVA showed a significant effect of the cognitive load, with a lower performance in the high cognitive load condition (*F*_(1,19)_ = 37.45, *p* < 0.001, $\eta_{p}^{2}$ = 0.66). The perceptual load had a near significant impact (*F*_(1,19)_ = 3.74, *p* = 0.06, $\eta_{p}^{2}$ = 0.16). The interaction term was not significant (*F*_(1,19)_ = 0.00, *p* > 0.05, $\eta_{p}^{2}$ = 0.00).

### Effects of Cognitive and Perceptual Loads on Ocular Behavior

**Figure 5:**
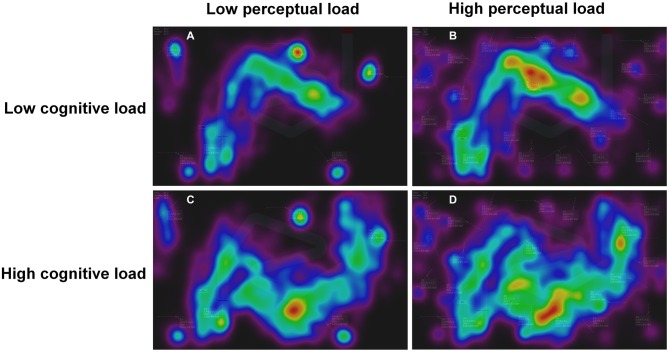
**Heatmap visualizations of the distribution of fixations on the Laby interface. (A)** Low cognitive load/low perceptual load; **(B)** low cognitive load/high perceptual load; **(C)** high cognitive load/low perceptual load; **(D)** high cognitive load/high perceptual load.

The analysis of the dispersion of fixations across the Laby interface is shown in Figure 5. In the high cognitive load condition, there is an increase in the overall time spent fixating the central part of the Laby interface where the central aircraft are moving. Also, the time spent fixating peripheral aircraft is increased in the high perceptual load condition.

**Figure 6:**
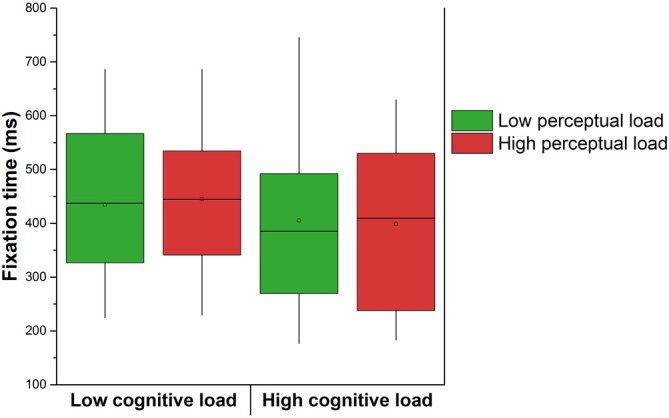
**Effect of the cognitive and perceptual loads on the fixation time averaged across the whole condition duration.** The square in the center of the boxes represent the mean, the horizontal line in the center of the boxes represent the 50th percentile (median), the end of the boxes represent the 25th and 75th percentiles, and the whiskers represent the 5th and 95th percentiles.

We analyzed the extent to which the overall fixation time, averaged across each whole condition duration, were affected by cognitive and perceptual loads (Figure 6). The 2 × 2 (cognitive load × perceptual load) showed a significant effect of cognitive load on fixation duration (*F*_(1,19)_ = 7.69, *p* < 0.05, $\eta_{p}^{2}$ = 0.29). The effect of the perceptual load was not significant (*F*_(1,19)_ = 0.00, *p* > 0.05, $\eta_{p}^{2}$ = 0.00) neither the interaction term (*F*_(1,19)_ = 0.41, *p* > 0.05, $\eta_{p}^{2}$ = 0.02). Overall average fixation durations were approximatively 420 ms (see Figure 6), which is consistent with a previous study using the same eye tracker during a simulated combat control system microworld. In this latter work, participants demonstrated average fixation durations above 300 ms in several experimental conditions (Hodgetts et al., 2015).

Fixation durations before a saccade have been shown to be modulated by the relative angle of the saccade (see, Wilming et al., 2013). The alternation between the two central planes in the high load condition could lead to systematic differences in the angle between subsequent saccades in comparison to the low load condition with only one central plane. This difference in angle by means of saccadic momentum can in turn lead to differences in fixation duration. Consequently, we compared the average angle between two saccades in the low vs. high cognitive load condition in order to examine a possible effect of momentum on fixation times. This analysis revealed that the mean angle slightly increased with increased cognitive load (low cognitive load = 84.64° (SD = 0.81); high cognitive load = 85.95° (SD = 1.08)) but the analysis did not reach the significance threshold (*F*_(1,19)_ = 2.24, *p* > 0.05, $\eta_{p}^{2}$ = 0.10). Saccadic momentum cannot explain by itself the variations of fixation times across the two cognitive load conditions.

### Effects of Cognitive and Perceptual Loads on the Inattentional Deafness Rate

**Figure 7:**
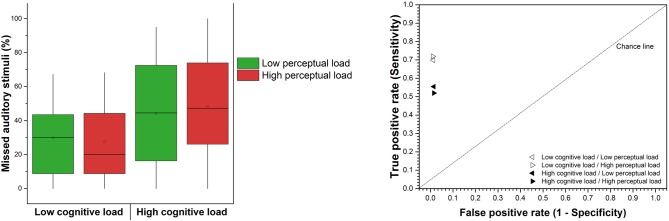
**Left:** mean percentage of missed auditory stimuli according to the levels of cognitive and perceptual loads (referred to as inattentional deafness rate). The square in the center of the boxes represent the mean, the horizontal line in the center of the boxes represent the 50th percentile (median), the end of the boxes represent the 25th and 75th percentiles, and the whiskers represent the 5th and 95th percentiles. **Right:** true positive rate vs. false positive rate according to the levels of cognitive and perceptual loads.

The control condition revealed that the inattentional deafness rate (missed alerts = 1-hit rate) was extremely low, with 2% (SD = 5.93) of missed alert. As a matter of fact, two participants omitted a few deviant tones whereas the 18 others reacted to 100% of the deviant tones. This result confirms that the tones were clearly perceptible despite the continuous white noise. We then examined if the inattentional deafness rate depended on cognitive and perceptual loads. The 2 × 2 (cognitive load × perceptual load) repeated measures ANOVA showed a significant effect of the cognitive load on the percentage of missed auditory alarms with an increased percentage of missed auditory stimuli in the high cognitive load condition (*F*_(1,19)_ = 24.49, *p* < 0.001, $\eta_{p}^{2}$ = 0.56), see Figure 7. The perceptual load had no significant impact (*F*_(1,19)_ = 0.10, *p* > 0.05, $\eta_{p}^{2}$ = 0.00). The interaction term was not significant (*F*_(1,19)_ = 0.49, *p* > 0.05, $\eta_{p}^{2}$ = 0.02). We finally examined if the specificity (true negative rate rate) and the discriminability index (d′ = Z(hit rate) − Z(false alarm rate); Stanislaw and Todorov, 1999) depended on cognitive and perceptual loads. A first 2 × 2 (cognitive load × perceptual load) repeated measures ANOVA revealed no significant main effect of cognitive load (*F*_(1,19)_ = 1.23, *p* > 0.05, $\eta_{p}^{2}$ = 0.06) and perceptual load (*F*_(1,19)_ = 0.07, *p* > 0.05, $\eta_{p}^{2}$ = 0.00) neither interaction effect (*F*_(1,19)_ = 0.22, *p* > 0.05, $\eta_{p}^{2}$ = 0.01) on the specificity. A second 2 × 2 (cognitive load × perceptual load) repeated measures ANOVA revealed significant main effect of cognitive load (*F*_(1,19)_ = 18.88, *p* < 0.001, $\eta_{p}^{2}$ = 0.50) but no effect of perceptual load (*F*_(1,19)_ = 0.00, *p* > 0.05, $\eta_{p}^{2}$ = 0.00) neither interaction (*F*_(1,19)_ = 3.30, *p* > 0.05, $\eta_{p}^{2}$ = 0.14) on the discriminability index. In summary, the cognitive load had a specific impact on the sensibility (hit rate), which increased the number of missed alerts. This is illustrated in Figure 7, the cognitive load had a specific impact on the true positive rate, not on the false positive rate. The d′ variations are only due to this effect of cognitive load on the true positive rate. We finally estimated if the variations in loads impacted the reaction time to alerts. In all four experimental conditions, mean reaction times were markedly below the mean time available to respond between two tones (i.e., 4.2 s), *M* = 1.67 s in low cognitive load condition; 1.53 s in high cognitive load condition; 1.65 s in low perceptual load condition; 1.55 s in the high perceptual load condition. The 2 × 2 repeated measures ANOVA showed no significant effect of cognitive (*F*_(1,19)_ = 1.02, *p* > 0.05, $\eta_{p}^{2}$ = 0.05) and perceptual load (*F*_(1,19)_ = 1.03, *p* > 0.05, $\eta_{p}^{2}$ = 0.05) neither significant interaction (*F*_(1,19)_ = 0.06, *p* > 0.05, $\eta_{p}^{2}$ = 0.00) on reaction times.

### Multivariate Logistic Regression Analysis

**Figure 8:**
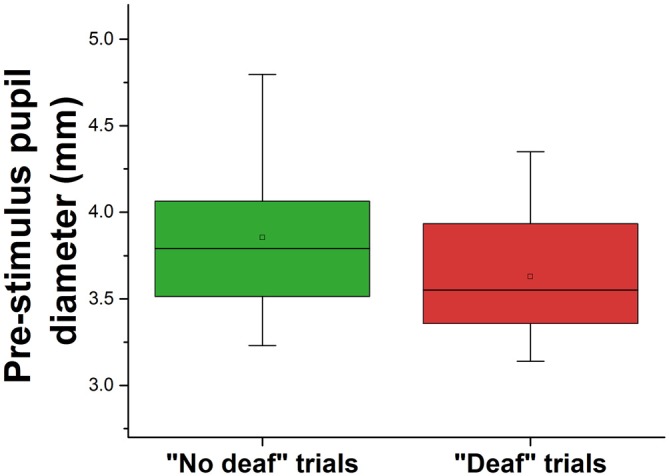
**Pre-stimulus pupil diameter (fixation *n*—1) for hit and missed auditory stimuli.** The square in the center of the boxes represent the mean, the horizontal line in the center of the boxes represent the 50th percentile (median), the end of the boxes represent the 25th and 75th percentiles, and the whiskers represent the 5th and 95th percentiles.

In order to further investigate the factors that promote inattentional deafness, we performed a multivariate logistic regression analysis stratified by trial (trials with deviant tones only) with all participants grouped together (number of trials = 800). We used the occurrence of inattentional deafness (yes/no) as binary dependent variable and cognitive and perceptual load were introduced as categorical independent variables. Furthermore, the mean duration of the fixation that occurred just before the occurrence of the rare tone and the mean pupil diameter during this fixation were used as a continuous variable. The Wald chi-square *p*-values confirmed that the cognitive load was a significant predictor of the occurrence of inattentional deafness (Wald statistic = 14.38, *p* < 0.001) while perceptual load was not (Wald statistic = 0.77, *p* > 0.05). In addition, the regression also showed that pupil diameter of the fixation that preceded the rare sound was significantly lower in the trials in which the participants did not react to the target tones (Wald statistic = 18.66, *p* < 0.001) (see Figure 8). Finally, the duration of the previous fixation was not predictive (Wald statistic = 0.20, *p* > 0.05).

## Discussion

### Summary of Results

Our results showed that a high level of cognitive load, manipulated by the number of planes to guide, significantly increased the inattentional deafness rate whereas the perceptual load, manipulated by the number of peripheral aircraft to monitor, had no significant impact. The cognitive load also impacted ocular behavior with lower fixation time in the high load condition, while perceptual load had no significant effect. Finally, logistic regressions showed that the mean pupil diameter of the fixation that preceded the onset of the tones predicted inattentional deafness.

### Effects of Cognitive and Perceptual Loads

Participants missed 28.76% of alarms in the low cognitive load condition (irrespective of the perceptual load) compared to 46.21% in the high cognitive load condition. This strikingly high rate of missed alerts cannot be attributed to sensorial difficulties as only 2% of alerts were missed in the control condition, in which the participant completed the tone detection task only (only 2 out of 20 participants missed any alerts). In the high cognitive load condition, participants engaged in greater mental effort by shifting their attention from one plane to the other, and also had a higher workload due to the need to calculate/modify heading and to change altitude parameters more often. These factors increased the chance of experiencing inattentional deafness.

Interestingly, perceptual load did not increase the missed alarm rate. In order to disentangle cognitive from perceptual load as much as possible, the high perceptual load did not generate a supplementary cognitive effort as the number of peripheral aircraft was increased while the number of associated peripheral alerts remained constant. Indeed, as demonstrated by Manning et al. (2001), the total conflict alert displayed contribute to increase cognitive effort. Inattentional deafness seems to be produced when an individual is engaged and monopolized in a task rather than when the individual is gazing more passively at visual information. One might argue that the additional number of peripheral aircraft was simply ignored by the participant which may explain this lack of effect of the high perceptual load condition. However, the heatmap illustrating the distribution of fixations clearly showed that fixations on the peripheral aircraft increased in the high perceptual load condition.

As demonstrated by the heatmaps and by a decline in the detection rate of peripheral notifications, the high load condition resulted in an important focus on the central aircraft, a behavior that can be compared to attentional tunneling (Wickens and Alexander, 2009; Régis et al., 2014). In general, fixation duration is known to reflect the attention (Findlay and Gilchrist, 2003) and mental effort (De Rivecourt et al., 2008) of an observer. In this last study on simulated flights, De Rivecourt et al. (2008) showed that momentary altitude changes can result in increased mean fixation duration. Variation of the fixation duration should be considered as task dependent: both shorter and longer fixations may indicate an increase in workload, and in particular shorter fixations indicated higher workload and increased temporal pressure in our study. This strong engagement of cognitive resources seemed to contribute to create a momentarily “deafness” to auditory stimuli.

One might also argue that this high inattentional deafness rate was due to an insufficient time window to report the alarm (i.e., mean time window = 4.2 s). Yet, in all four experimental conditions, mean reaction times were markedly below the mean time available to respond between two tones (around 1 s), and the reaction times did not significantly vary across the four experimental conditions. Even if we cannot completely eliminate the idea that a relatively small number of deviant tones were not reported because participants reacted too late, these two results tend to exclude this explanation as a major contributor of variation in the inattentional deafness rate with increased cognitive load. The analysis of d′ confirmed that the decline in the number of reported alerts in the high cognitive load condition is associated with a loss of sensitivity to deviant tones, and not due to an effect on the ability to discriminate the two tones. In this latter situation, the number of false alarms would have likely increased.

Importantly the inattentional deafness rate of the present study was considerably higher than in previous research using Laby (Giraudet et al., 2015a), whereby the percentage of unreported tones was 6% in the high cognitive load condition. To further understand the factors that promote the occurrence of inattentional deafness, the present study had employed two modifications to create a more engaging and complex version of the Laby task. First, there were two aircraft to guide in the high cognitive load condition whereas only one was displayed in the previous study. Second, in the current study participants had to mentally calculate the various headings that the aircraft should follow to turn and stay within the corridor, whereas previously these were pre-calculated by the system and just required selection from a drop-down menu. These modifications lead to a considerable rise in the incidence of inattentional deafness. It must be noted that in both studies, the importance of reporting the sounds was emphasized and that the time between the two tones was identical in both. The mental calculation of heading was undoubtedly a key factor in this increase of inattentional deafness as even in the low load condition, in which only one aircraft was displayed, the inattentional deafness rate was greater than three times that observed in the previous study in which the heading was given by the system.

### Lower Pupil Diameter Predicts Inattentional Deafness

The multivariate logistic regression confirmed that cognitive load significantly predicted the occurrence of inattentional deafness. Most importantly, the regression also revealed that pupil diameter was lower during the fixation that preceded the onset of the target tones in the “deaf” trials. This result is counterintuitive as inattentional deafness was indubitably increased by the high cognitive load context, which is supposed in turn to increase the pupil diameter (Kahneman and Beatty, 1966; Palinko et al., 2010; Peysakhovich et al., 2015). Yet, as previously mentioned, Beatty (1982) showed that vigilance decrement was associated to decreased amplitude of the phasic task-evoked pupillary response during an auditory vigilance task, while tonic or baseline pupillary diameter exhibited no such relationship. In addition, a very recent study (Unsworth and Robison, 2016) indicated that pupillary diameter can index lapses of sustained attention. They showed that compared to focused states, inattentive and mind-wandering states are associated with lower pretrial baseline pupil responses and that distracted states are associated with larger pretrial baseline pupil diameter. These results support the notion that pupil diameter is sensitive to different types of lapses of attention, which is consistent with theories of locus coeruleus norepinephrine (LC-NE) functioning. In our study, despite a context of sustained high cognitive load, momentary lapses of sustained attention may have occurred, which could explain the relationship between lower phasic pupil diameter and inattentional deafness occurrence. This assumption can be also related to a past study that revealed that information overload resulted in a leveling of the dilation pattern, which suggested a momentary suspension of processing effort (Peavler, 1974).

Our results demonstrating an effect of cognitive load but not of perceptual load on inattentional deafness, are somewhat contradictory to a study by Macdonald and Lavie (2011) in which participants were engaged in a visual discrimination task of a cross shape. In the low visual load condition, this discrimination was made according to the line color and in the high visual-load condition, participants had to discriminate subtle line length. One brief pure tone was presented simultaneously at the final trial onset. Failures to notice the presence of this tone reached a rate of 79% in the high-visual-load condition, significantly greater than in the low-load condition. We could postulate that the type of perceptual load manipulated by the authors likely generated an indirect increase in mental effort and task engagement due to the comparison process of the line length. For example, Fierro et al. (2000) showed that the line-length comparison process engages the parietal cortex, indicating that spatial cognition is also taxed in such a task. Also, the paradigm used by Macdonald and Lavie (2011) was quite different as only one pure sound was presented in the study while 10 target tones per condition were presented in the current study. We believe that our paradigm is closer to a real life context or complex activity such as ATC in which the auditory environment is composed of a mixture of different sounds that can be repeated several times. As our paradigm can be related to “change deafness” studies, for example in which a subtle change between two voices is unnoticed (Vitevitch, 2003), a future study would look to reproduce the same paradigm but with only deviant tone.

### Conclusion

The present study suggests that inattentional deafness is promoted by cognitive load rather than by a “passive” perceptual load that does not generate a supplementary amount of work. A strong engagement of cognitive resources in a given task can momentarily render one “deaf” to auditory stimuli. In our study, the key factor that promoted inattentional deafness was most likely the cognitive load generated by the mental calculation of heading and by the numerous tasks to conduct. This result confirmed previous studies showing that inattentional deafness drastically increases in the context of high cognitive load (Giraudet et al., 2015b), which can have serious consequences in safety-critical occupations like ATC. Finally, the mean pupil diameter of the period that just preceded the rare sound onset was significantly lower in the trials in which the participants did not react to the target tones, likely showing a momentary lapse of sustained attention, which in turn promoted the occurrence of inattentional deafness.

## Ethics Statement

The study was approved by the National Scientific Research Ethics Committee in Paris (CEEI/IRB00003888).

## Author Contributions

LG, J-PI and MC designed the experiment. LG conducted the experiments. LG, MC, J-PI analyzed and interpreted the data. MC, LG, ST and CJ wrote the article.

## Funding

Financial support was provided by a Discovery grant from the National Sciences and Engineering Research Council of Canada (NSERC), awarded to Sébastien Tremblay [Grant number CG073877], by the Institut Supérieur de l’Aéronautique et de l’Espace to in the form of an operating grant to Mickaël Causse, and also by the Direction Générale de l’Armement in the form of a scholarship to Louise Giraudet.

## Conflict of Interest Statement

The authors declare that the research was conducted in the absence of any commercial or financial relationships that could be construed as a potential conflict of interest.
